# Do Bovine Lymphocytes Express a Peculiar Prion Protein?

**DOI:** 10.1080/10446670310001593550

**Published:** 2002-12

**Authors:** France Mélot, Caroline Thielen, Thouraya Labiet, Sabine Eisher, Olivier Jolois, Ernst Heinen, Nadine Antoine

**Affiliations:** Laboratory of Human Histology (Professor Ernst Heinen) Centre de recherche prion University of Liège, 20 rue de Pitteurs Liège B-4020 Belgium

## Abstract

The cellular prion protein (PrPc) is a glycolipid-anchored cell surface protein that usually exhibits three glycosylation states. Its post-translationally modified isoform, PrPsc, is involved in the pathogenesis of various transmissible spongiform encephalopathies (TSEs). In bovine species, BSE infectivity appears to be restricted to the central nervous system; few or no detectable infectivity is found in lymphoid tissues in contrast to scrapie or variant CJD. Since expression of PrPc is a prerequisite for prion replication, we have investigated PrPc expression by bovine immune cells. Lymphocytes from blood and five different lymph organs were isolated from the same animal to assess intra- and interindividual variability of PrPc expression, considering six individuals. As shown by flow cytometry, this expression is absent or weak on granulocytes but is measurable on monocytes, B and T cells from blood and lymph organs. The activation of the bovine cells produces an upregulation of PrPc. The results of our in vitro study of PrPc biosynthesis are consistent with previous studies in other species. Interestingly, western blotting experiments showed only one form of the protein, the diglycosylated band. We propose that the glycosylation state could explain the lack of infectivity of the bovine immune cells.


*Developmental Immunology*, December 2002 Vol. 9 (4), pp. 245–252

Taylor & Francis healthsciences

### Do Bovine Lymphocytes Express a Peculiar Prion Protein?*

FRANCE MÉLOT, CAROLINE THIELEN, THOURAYA LABIET, SABINE EISHER, OLIVIER JOLOIS, ERNST HEINEN and NADINE ANTOINE†

*Laboratory of Human Histology (Professor Ernst Heinen), Centre de recherche prion, University of Liège, 20, rue de Pitteurs, B-4020, Liège, Belgium*

The cellular prion protein (PrPc) is a glycolipid-anchored cell surface protein that usually exhibits three glycosylation states. Its post-translationally modified isoform, PrPsc, is involved in the pathogenesis of various transmissible spongiform encephalopathies (TSEs). In bovine species, BSE infectivity appears to be restricted to the central nervous system; few or no detectable infectivity is found in lymphoid tissues in contrast to scrapie or variant CJD. Since expression of PrPc is a prerequisite for prion replication, we have investigated PrPc expression by bovine immune cells. Lymphocytes from blood and five different lymph organs were isolated from the same animal to assess intra- and interindividual variability of PrPc expression, considering six individuals. As shown by flow cytometry, this expression is absent or weak on granulocytes but is measurable on monocytes, B and T cells from blood and lymph organs. The activation of the bovine cells produces an upregulation of PrPc. The results of our *in vitro* study of PrPc biosynthesis are consistent with previous studies in other species. Interestingly, western blotting experiments showed only one form of the protein, the diglycosylated band. We propose that the glycosylation state could explain the lack of infectivity of the bovine immune cells.

*Keywords*: Prion protein; Bovine lymphocytes; Glycosylation; Flow cytometry; Western blotting

### INTRODUCTION

Transmissible spongiform encephalopathies (TSEs) are neurodegenerative diseases including bovine spongiform encephalopathy (BSE), scrapie, Creutzfeldt-Jakob disease (CJD) and variant (vCJD), fatal familial insomnia, Gerstmann-Sträussler-Scheinker's syndrome and Kuru. The principal marker of those illnesses is a protein called PrPsc; its core (PrPres) resists to treatment with proteases such as proteinase K (Prusiner, 1982). The cellular form of the prion protein (PrPc) is currently thought to be the substrate for the synthesis of its pathogenic counterpart (Bueler *et al.*, 1993). PrPc is encoded in the genome of all mammals investigated to date and seems to have an essential but not yet defined function as shown by a high degree of evolutionary conservation (Krakauer *et al.*, 1998). In the central nervous system, PrPc expression is very high and correlates with an important accumulation of infectivity in the TSEs (Wells *et al.*, 1994). In comparison, all the other organs have a much lower expression of PrPc (Fournier, 2001). Upon peripheral administration of the agent, accumulation and/or replication of the prion particles is first found in the spleen (McBride *et al.*, 1992). Different cells of the immune system appear implicated in the pathogenesis: lymphocytes, Follicular dendritic cells (FDCs), macrophages and dendritic cells (Klein *et al.*, 1997; Brandner *et al.*, 1999; Brown *et al.*, 1999; Montrasio *et al.*, 2000; Beringue *et al.*, 2000; Huang *et al.*, 2002). FDCs could act as key players, B lymphocytes and other immune cells having an indirect role as shown by the different studies using immunodeficient mice (Klein *et al.*, 1998), or knock-out mice for the lymphotoxin or TNF pathways (Mabbott *et al.*, 2000;2002; Prinz *et al.*, 2002).

BSE epidemy and the concomitant emergence of the variant of the CJD in humans (vCJD) raised different questions. In fact, the numerous common features between both agents made inter-species transmission of BSE to human being considered as possible (Collinge, 1997; Scott *et al.*, 1999). Tissue infectivity in BSE has thus been comprehensively investigated in cattle with natural or experimental BSE (Mohri *et al.*, 1992; Somerville *et al.*, 1997; Wells *et al.*, 1998; Pammer *et al.*, 1999; Bradley, 1999; Race *et al.*, 2000). In the case of the BSE, several researches reported lack of accumulation of the infectious agent within the lymphoid organs, excepted the Peyer's patches of the distal ileum (Wells *et al.*, 1994; Farquhar *et al.*, 1996; Wells *et al.*, 1998). This peculiarity of the bovine immune system is intriguing. As PrPc is the substrate for the development of infectivity, different level of expression could affect the infection and the propagation of the disease. However, knowledge about the expression of PrPc by the different lymphoid populations in different organs is limited.

*Presented at the Proceedings of the 4th Germinal Center Conference, June 2002, Groningen, The Netherlands.
†Corresponding author. Tel.: +32-4-366-51-85. Fax: +32-4-366-51-73.

ISSN 1044-6672 print © 2002 Taylor & Francis Ltd
DOI: 10.1080/10446670310001593550

In this work, we report the investigation of the PrPc expression at the surface of bovine lymphoid cells by flow cytometry. Cytoplasmic and surface content in PrPc of bovine lymphocytes as well as their isoform distribution were then determined by western blotting. We also studied *in vitro* the modulation of PrPc expression, reflecting biosynthesis under different *in vivo* conditions: enzymatic treatment, stimulation with mitogens and blockade of glycosylation.

### MATERIALS AND METHODS

### Materials

Blood, palatine tonsils, spleens, Peyer's patches, thymus and lymph nodes were obtained at a local abattoir from clinically healthy 18 months old cattle. Organs were placed in phosphate buffer saline (PBS) and kept at 4°C until they were processed for isolation of lymphoid cells. Special attention was dedicated to collect the blood and organs from the same individual in order to assess the inter- and intra- individual variability of PrPc expression.

All reagents were of analytical grade and were acquired from Sigma (Belgium) unless specifically mentioned. Antibodies against bovine lymphoid subpopulations were purchased from Serotec, UK.

Antibodies against PrP (SAF34, 12F10 at a concentration of 1 mg/ml) were obtained from Dr J. Grassi, CEA, France. SAF34 antibody was produced by the team of Dr J. Grassi against AA79-92 whereas 12F10 antibody was raised against AA142-160 by the team of Dr Gerhard Hunsmann.

### Isolation of Peripheral Blood Mononuclear Cells

Lymphoid cells from complete peripheral blood were separated by density centrifugation on Ficoll-Paque (Amersham Pharmacia, Sweden). Lymphocytes and monocytes were recovered from the interphase, washed four times with PBS and then resuspended in RPMI 1640 (Bio-Whittaker, Belgium).

The granulocytes were recovered after complete blood was submitted to red blood cells lysis with water.

### Isolation of Tonsil, Spleen, Peyer's Patch, Thymus and Lymph Node Lymphocytes

The different bovine organs were cut into fragments, disrupted and filtered through a 150 μm nylon filter to eliminate aggregates and membrane fragments. The filtrate containing lymphoid cells was separated by density centrifugation on Ficoll-Paque. Lymphocytes and monocytes were recovered from the interphase, washed four times and then resuspended in RPMI 1640.

### Analysis of PrPc Expression by Flow Cytometry

SAF34 or 12F10 mouse Mab recognizing distinct prion protein regions were used in indirect labeling experiments. Different lymphocyte subpopulations are discriminated by a staining with CD4, CD8 or CD21 specific Mab.

Briefly, $10^6$ cells were first incubated in PBS for 30 min at 4°C with FITC-coupled antibodies (CD4 or CD8 1/500) or with antibody against CD21 (1/200) followed by FITC-coupled rabbit anti-mouse Ig (Dako, Denmark 1/1000). The cells were washed, and then incubated for another 30 min in PBS with biotinylated SAF34 or 12F10 mouse Mab (diluted 1/1000), washed, incubated for 30 min in PBS with PE-conjugated streptavidin (diluted 1/2000, Becton-Dickinson, California) and finally rinsed before analysis with a FACScalibur (Becton-Dickinson, California). Cells incubated without specific MAbs were used as negative controls. The regions containing lymphocytes, monocytes and granulocytes were discriminated on the basis of the cell size (FSC) and granularity (SSC) showed on the dotplot. The region containing the lymphocytes was then further investigated on a dotplot of the fluorescent signals: FL1 represents the labeling of each subpopulation, the staining of the prion protein is reported onto the FL2 axis. Quadrant statistics were given by the Cell Quest program. The results are expressed as the percentage of double-positive cells within each cell subpopulation.

### Cell Cultures

For all experiments lymphoid cells were grown in RPMI 1640 supplemented with 10% FCS, 2 mM L-glutamine, 100 μg/ml penicillin and streptomycin. In every condition, cells were counted and their viability was assessed by trypan blue exclusion test with a survival of approximately 95–98%.

In order to test the role of the activation status on the PrPc expression of the cells, the lymphocytes from spleen or mesenteric lymph nodes were incubated either with or without different mitogens as L-phytohaemagglutinin (PHA-L; 1 or 2 μg/ml), or phorbol myristate acetate (5 or 10 ng/ml) and ionomycin (0.5 or 1 μg/ml) (PMA-iono) during 6, 24 or 48 h at 37°C with 5% $CO_2$. The efficiency of the treatment was monitored by $^3$H thymidine (T$^3$H) incorporation as in Takeda *et al.* (1996) to measure proliferative responses of mononucleated cells.

### Digestion of PrPc at the Cell Surface

We have treated the freshly isolated cells with an enzymatic cocktail containing 1 mg/ml collagenase A, 0.5 mg/ml dispase type II, 0.04 mg/ml DNase type I (Boerhinger-Mannheim, Germany) in RPMI 1640 with 0.4% BSA during 1 h at 37°C. The efficiency of the digestion of PrPc from the cell surface was monitored by cytometric analysis after labeling with the SAF34 Mab.

### Blockade of Glycosylation

To analyze the capacity of the cells to display the unglycosylated protein at the surface of the membrane, we stopped the protein glycosylation process. Cells were thus incubated with tunicamycin after the enzymatic digestion. The incubation was realized either with or without tunicamycin at a final concentration of 1, 2 or 5 μg/ml, during 18 h at 37°C with 5% $CO_2$.

### Analysis of PrPc by Western Blotting

Immediately after isolation or culture, the cells were quickly processed for the SDS-Page and the samples were frozen at −20°C. Briefly, $10^6$ cells were peletted, resuspended in 20 μl of Tris migration buffer (containing 120 mM Tris–HCl, 60 mM sucrose, 70 mM sodium dodecylsulfate, 0.15 mM bromophenol blue, 0.5% 2-mercaptoethanol and 1% Triton X-100 pH 6.8), passed five times trough a 22 1/4 gauge-needle and boiled during 2 min. The brain sample was prepared by homogeneizing 100 mg of tissue into 1 ml of buffer (Tris/HCl 10 mM pH 7.4, 100 mM NaCl, 10 mM EDTA, 0.5% NP-40, 1% sodium deoxycholate). After centrifugation (10 min, 2500*g*), the supernatant was diluted 1/2 in 2 times concentrated Tris migration buffer. The cell or tissue homogenates were subjected to SDS-Page 12%. Proteins were transferred to a nitrocellulose membrane (Millipore, Bedford, USA) by electroblotting in a transblot (BioRad, USA) transfer apparatus for 1h at room temperature. Before the transfer, the gels, Whatman filter papers, and nitrocellulose membrane, were soaked in electroblotting buffer (25 mM Tris–HCl; 192 mM glycine; 20% methanol, pH 8.0) for 15 min. After transfer, the membrane was blocked using tris buffer saline (TBS) with 5% dry milk overnight at 4°C. The prion protein was detected by incubating the membrane for 1h at room temperature with mouse monoclonal antibody against prion (SAF 34) diluted 1:500. The membrane was washed three times in TBS with 0.1% Tween 20 (TBST). Specific protein was detected using a horseradish peroxidase-conjugated secondary antibody and enhanced chemiluminescence (ECL) reagents (Amersham, UK). Image analysis device was a ScanJet II scanner with DeskScan II software (Hewlett Packard, Palo Alto, CA, USA).

### RESULTS

### Flow Cytometry Analyses of PrPc Expression on Lymphocytes

We first analyzed the PrPc labeling in blood samples. Monocytes and lymphocytes expressed the PrPc at their surface while granulocytes were globally negative for the PrPc immunolabeling (Table I).

The staining of surface PrPc was then realized in parallel on freshly isolated blood, spleen, tonsils, Peyer's patches, thymus and lymph nodes cells. These results were analyzed for six animals. Figure 1 shows the percentage of PrPc positive cells in each subpopulation: CD4 (panel *a*), CD8 (panel *b*) and CD21 (panel *c*). If we consider the same organ, approximately the same levels of cell surface staining were observed respectively for each cell population with the two PrP-specific antibodies (12F10 gives slightly minor values, data not shown).

TABLE I Expression of PrPc on freshly isolated blood mononuclear cells (as a percentage of total cell population): cytofluorimetric analysis with the SAF34 Mab. $n = 6$

| Blood cell population | Granulocytes | Monocytes | Lymphocytes |
|---|---|---|---|
| Percentage of PrPc expression | 0.8 ± 0.7% | 31.8 ± 3.4% | 63.2 ± 7.6% |

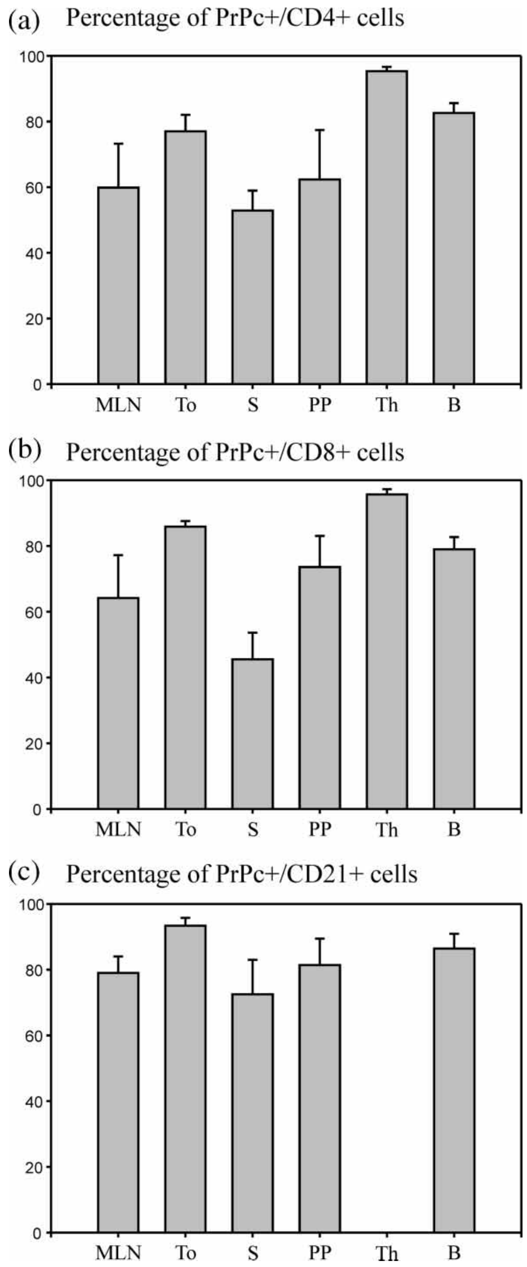

FIGURE 1 Expression of PrPc on the bovine lymphoreticular system cells (as a percentage of total cell population): cytofluorimetric analysis on freshly isolated mononuclear cells subpopulations from blood (B), spleen (S), tonsils (To), thymus (Th), Peyer's patches (PP) and mesenteric lymph nodes (MLN) with the SAF34 Mab. $n = 6$.

The amount of PrPc expressed at the surface of thymic or blood T lymphocytes was consistently more important than on spleen lymphocytes while it is intermediate on the lymph nodes, tonsils and Peyer's patches lymphocytes. This situation appears less marked in the case of the B cells. For those cells the mean labeling percentages are a little higher, specially in the case of the tonsils.

An important variability in the percentage of positively stained splenic cells was noticed reflecting important interindividual variations in prion protein expression. The staining intensities were also reduced within the spleen (data not shown).

### Effect of Activation on PrPc Level

In order to test the influence of activation, spleen and lymph node lymphocytes were maintained in culture conditions with or without mitogens. We showed that bovine splenic and mesenteric lymph node lymphocytes are able to express high quantities of PrPc at their surface in culture conditions even without stimulation (Fig. 2 (b)). An increase in [$^3$H] thymidine incorporation, reflecting the DNA synthesis due to cell proliferation, was seen after 6, 24 or 48 h of treatment with PHA or PMA/ionomycin.

High stimulation was obtained after 48 h of treatment with PMA/ionomycin. A representative histogram out of three experiments is shown on Fig. 2 (b). Cultured splenic lymphocytes demonstrated an increased cell volume (Fig. 2(a)) and a higher PrPc expression (Fig. 2 (b)). The lymphocytes from the mesenteric lymph nodes showed a minor increase of the PrPc staining (data not shown).

### Cleavage of Cell Surface PrPc

A 1 h digestion with an enzymatic cocktail containing collagenase-dispase-DNase abolished the surface PrPc labeling monitored by FACS analysis. Reexpression of the membrane protein *in vitro* was observed when digestion was followed by a short-term culture. After 6 h of culture, we detected a slight increase of the signal in treated cells (Fig. 3 (a) and (b)). After 18 h of culture, the PrP was totally reexpressed at the surface of the membrane by the treated cells.

### PrPc Western Blotting

For all immune cells, western blotting with the SAF34 Mab (Fig. 4) revealed only one form of PrPc. The unique band within all the lymph organs was located at the same molecular weight as the diglycosylated band from the brain (34 kDa). The brain isoforms were located at 29, 31 and 34 kDa. A small isoform was found in the spleen and blood, and had a molecular weight of 19 kDa, certainly corresponding to a truncated form.

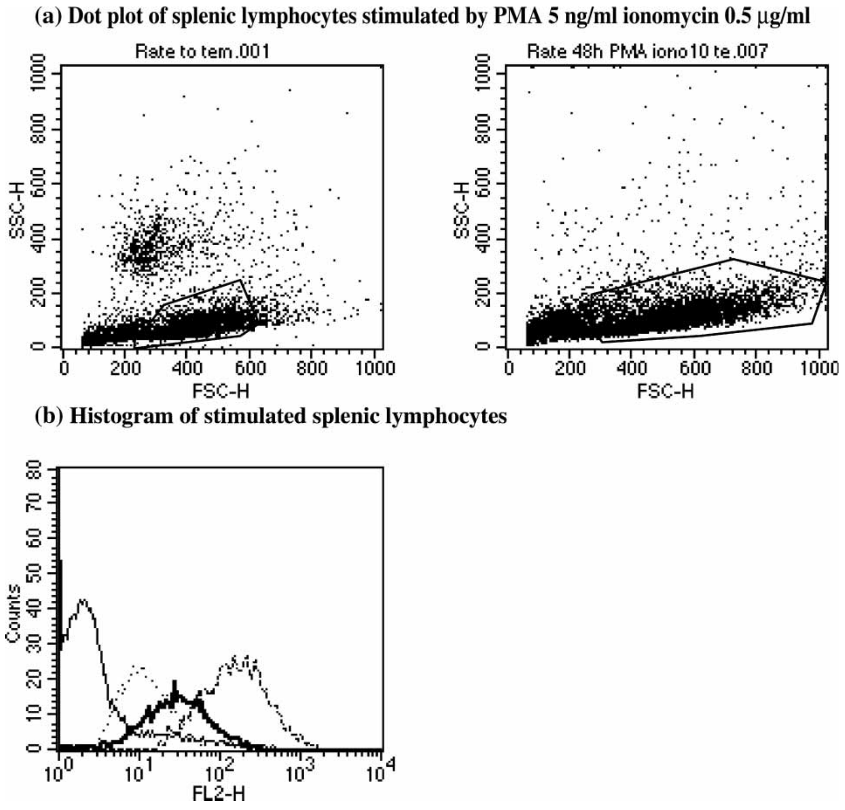

FIGURE 2 Comparative dot plots of splenic lymphocytes directly after isolation and 48 h of culture with mitogen (a). Histograms of PrPc expression by spleen lymphocytes (b): the effect of stimulation by phorbol myristate acetate in combination with ionomycin. The lymphocytes were stained directly after isolation (dotted line histogram) or after 48 h of culture with (dashed line histogram) or without mitogen (bold line histogram). Cells were stained indirectly with SAF34 Mab and rabbit anti-mouse Ig PE-conjugated secondary antibody. Control = secondary antibody alone (normal line histogram). $n = 3$.

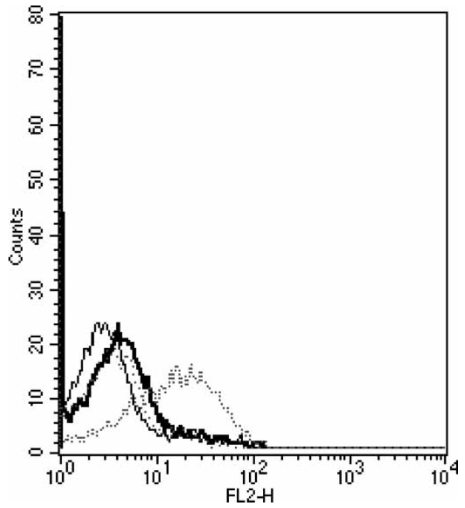

FIGURE 3 Comparative histograms of PrPc expression on enzymatically treated splenic lymphocytes directly after isolation (bold line histogram) or after six (dotted line histogram) or 18 h (dashed line histogram) of culture. Cells were stained indirectly with SAF34 Mab and rabbit anti-mouse Ig PE-conjugated secondary antibody. Control = secondary antibody alone (normal line histogram). $n = 3$.

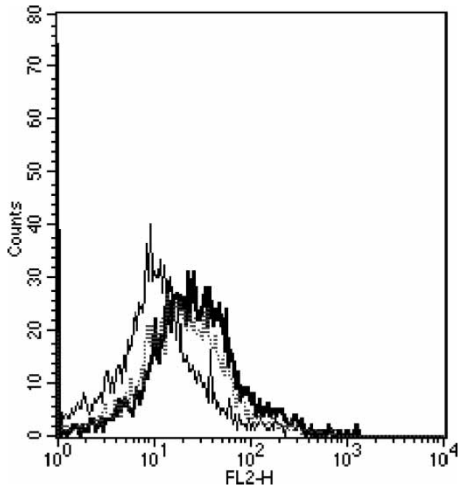

FIGURE 5 Comparative histograms of PrPc expression by enzymatically treated splenic lymphocytes directly after a 18 h incubation with (dashed line histogram) or without tunicamycin (bold line histogram). Cells were stained indirectly with SAF34 MAb and rabbit anti-mouse Ig PE-conjugated secondary antibody. Control = secondary antibody alone (normal line histogram). $n = 3$.

The labeling pattern was identical with the 12F10 Mab, nevertheless the band intensities were weaker.

### Modulation of PrPc Expression on Cultured Lymphocytes

After incubation with enzymes to destroy the PrPc present in the membrane, the cells isolated from the spleen or mesenteric lymph nodes were treated *in vitro* with tunicamycin which prevents lipid-carrier dependent N-glycosylation of proteins. Concentrations of 1, 2 or 5 μg/ml were used since higher doses (30 and 50 μg/ml) were shown by others to inhibit protein synthesis. The efficiency of this treatment on lymphocytes was checked by western blot with the SAF34 Mab which revealed only the unglycosylated form of PrPc (29 kDa—data not shown). After treatment, PrPc was analyzed at the surface of B and T cells by flow cytometry. After an 18 h incubation with or without 5 μg/ml of tunicamycin, PrPc expression by spleen lymphocytes (Fig. 5) was equivalent in both conditions.

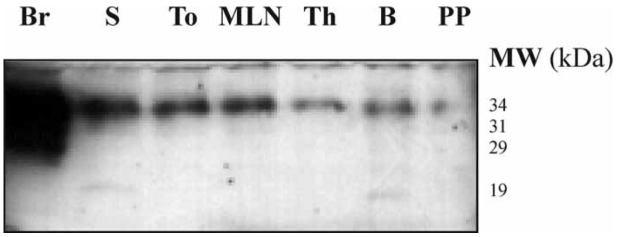

FIGURE 4 Western blot detection of PrPc with SAF34 MAb on brain (Br) and splenic (S), tonsilar (To), mesenteric lymph nodes (MLN), thymic (Th), blood (B) and Peyer's patch (PP) lymphocytes directly after isolation.

### DISCUSSION

Naturally occurring TSEs are probably transmitted by oral or other peripheral routes of infection. Following peripheral inoculation, the role of the immune cells was clearly established using murine models of scrapie neuroinvasion (Raeber *et al.*, 1998) and in humans suffering of vCJD (Hill *et al.*, 1999). In fact, defects affecting B-lymphocytes in mice, either at the level of the differentiation or of the capacity of response, prevented the development of clinical scrapie (Klein *et al.*, 1997). Race and collaborators (2000) debated the possibility of hematogenous diffusion via leukocytes as a mechanism of peripheral spread of infection to the brain. This situation contrasts with the lack of infectivity in non-neural tissues of natural and experimentally infected cattle, excepted in the distal ileum at 6 months post-exposure (Wells *et al.*, 1994; Farquhar *et al.*, 1996; Bradley, 1999). As PrPc expression is required for susceptibility to TSEs (Bueler *et al.*, 1993; Weissman *et al.*, 1994), this study was designed to analyze characteristics of PrPc expression at the surface of different bovine immune cells. The regulation of PrP expression in different immune cell populations would not only be important with regard to the normal cellular function of PrPc but also have relevance for the understanding of prion disease pathogenesis.

We observed by cytofluorimetry that, as previously shown in humans (Cashman *et al.*, 1990; Dodelet and Cashman, 1998; Barclay *et al.*, 1999; Antoine *et al.*, 2000; MacGregor *et al.*, 2000; Barclay *et al.*, 2002), PrP is expressed in blood by B and T cells and monocytes but not in significant amounts on granulocytes. A study by Dürig *et al.* (2000) demonstrated that the human blood monocytes have the same level of labeling at their surface as T cells, and that B cells express less PrPc. Our data show that bovine monocytes constitutively express less PrPc than T cells and B cells do. On the basis of analyses performed on immune cells prepared from different lymphoid tissues from the same animal and reproduced on six individuals, there was no evidence of any link between the lymphocyte subtype and the presence or amount of surface PrP. We demonstrated that less splenic cells are PrPc positive and that the staining is less intense compared to that of other organs. One should notice that for the spleen the data are globally the most variable. This parallels the study of mature splenic B and T cells in mice by Liu *et al.* (2001) or the study of ovine tonsils and spleen by Moudjou *et al.* (2001). The numerous splenic macrophages and granulocytes might release enzymes able to detach surface PrPc.

In human samples, the intensity of the staining observed for blood cells was high in comparison to that in the tonsils (Antoine *et al.*, 2000). This reduced intratissular PrPc expression by human lymphocytes may result from a reduced PrPc synthesis (down regulation) or may be due to enzymatic cleavage when lymphocytes interact with the extracellular matrix or with other cells (Jimenez-Huete *et al.*, 1998). We compared again the situation in human with our experimental conditions on bovine samples. We induced detachment of surface PrPc of living lymphocytes with an enzymatic cocktail containing coll-agenase-dispase-DNase after the data on the hydrolysis of nascent PrPc from the cell surface of neuroblastoma (N2a) cells with dispase (Borchelt *et al.*, 1990;1992). We demonstrated that following *in vitro* exposure to proteases, PrPc is reexpressed after a short-term incubation period which correlates with the approximately 3 to 6 h half-life measured on hamster and chicken PrPc anchored in the plasma membrane in neuroblastoma cells (Taraboulos *et al.*, 1992; Shyng *et al.*, 1993).

We showed that bovine splenic and mesenteric lymph node lymphocytes are able to express high quantities of PrPc at their surface in culture conditions even without stimulation. However, a treatment with PMA/ionomycin upregulated PrPc expression at the surface of the activated cells *in vitro*. Conflictual data were published as to the effect of mitogenic stimulation: different works (Cashman *et al.*, 1990; Mabbott *et al.*, 1997; Dürig *et al.*, 2000; Li *et al.*, 2001) suggest that PrPc expression on haematopoietic cells correlates with their activation and developmental status and that activation may play a role in the propagation of TSEs. Nevertheless, cultivated human tonsillar lymphocytes increase their PrPc expression independently of the mitogenic activation (Antoine *et al.*, 2000). Furthermore, human blood cells express more PrPc than within tissues but they are clearly less stimulated. A unifying hypothesis may be that activation enhances PrPc expression but that intercellular contacts (i.e. enzymatic cleavage of surface PrPc) or intracellular recycling induce the loss of surface PrPc.

We have previously detected PrPc with two antibodies (SAF34 and 12F10) recognising N- and C-terminal epitopes, respectively. Surprisingly, the results as to the location of this protein within the germinal center and more precisely on the FDCs depended on the antibody used. We hypothesized that FDCs in bovine organs express a particular form of PrPc. The N-terminal epitope of PrPc might be absent (truncated form) in those samples, or the sequence recognized by SAF34 MoAb might be masked by interaction with other molecules (Thielen *et al.*, 2001). In the present study, we could demonstrate that the antibodies SAF34 and 12F10 react with PrPc of different lymphocytes. This observation means that the PrPc present at the surface of the lymphocytes is not particular as to its N-terminal part. Moreover, the amount of PrPc detected at the surface of the lymphocytes was consistently less important with the 12F10 than with the SAF34.

In order to refine our analyses, we decided to reveal the total content and the isoform distribution of PrP in those cells by immunoblotting techniques. Since no small fragments were detected in most samples, our analyses confirm that the lymphocytes do not follow the particular PrPc pattern observed in FDCs. In light of this result, we confirm the hypothesis that the majority of lymphocytic PrPc is not truncated. The presence of only a diglycosylated band appears interesting. Expressed by immune cells, this diglycosylated isoform might perturb the conversion of PrPc into PrPres as shown on other models by Capellari *et al.* (1999). Anyway, different glycosylation patterns between neural and immune tissues or within different parts of the nervous system were already described in other species (Li *et al.*, 2001; Madec *et al.*, 2000; Russelakis-Carneiro *et al.*, 2002).

Tunicamycin treatments, realized at a concentration of 5 μg/ml, evidenced a highly reduced synthesis of the 31–34 kDa forms and an increase in the amount of the 29 kDa band which corresponds to the unglycosylated form, as reported in bovine spleen, kidney, liver and adrenal gland by immunoblotting with the mAb BSPX-54 (Horiuchi *et al.*, 1997). We could conclude from our flow cytometric data that in those conditions, N-glycosylation is not necessary for the perfect folding and trafficking of the bovine PrPc to the cell surface. This is in accordance with observations in other species (Petersen *et al.*, 1996; Lehmann and Harris, 1997).

Globally, our results support the notion that lymphocytes might represent a possible target for prion transport and replication in the bovine blood and lymphoid organs. As revealed by the increase of PrPc in blood and its decrease in tissues during lymphocyte recirculation, we can conclude on the basis of our observations on bovine and human (Antoine *et al.*, 2000) that in tissues, enzymatic cleavage of surface PrPc may explain its loss. Small differences in the surface distribution of electrostatic charges of bovine PrP, compared to that of the human prion protein (Lopez Garcia *et al.*, 2000), or interspecies variation of the immune system within mammals (Lascelles and McDowell, 1974; Gallo and Wong-Staal, 1982)

could explain the small discrepancies between previous results on human and the present study. Additional factors are thus required to explain the lack of infectivity of the bovine immune cells; the glycosylation state could be one of them. The key of this non-infectious character might also reside in the interaction of PrPc with other molecules.

### Acknowledgements

The prion protein antibodies were kindly provided by the Deutsche Primatenzentrum GMH represented by Prof. G. Hunsmann and W. Bodemer (MAb 3B5) and by the "Commissariat à l'énergie atomique", the CEA Saclay represented by Prof. J. Grassi and Y. Frobert (MAb SAF34). We also want to thank Mrs Mia Jackers for her excellent technical help. This study was supported by the European FAIR Contract (J-CT98-6022) and by the DGTRE of the "Région Wallonne".

### References

Antoine, N., Cesbron, J.Y., Coumans, B., *et al.* (2000) "Differential expression of PrPc on human blood and tonsil lymphocytes", *Haematologica* **85**(5), 475–480.

Barclay, G.R., Hope, J., Birkett, C.R. and Turner, M.L. (1999) "Distribution of cell-associated prion protein in normal adult blood determined by flow cytometry", *Br. J. Haematol.* **107**, 804–814.

Barclay, G.R., Houston, E.F., Halliday, S.I., Farquhar, C.F. and Turner, M.L. (2002) "Comparative analysis of normal prion protein expression on human, rodent, and ruminant blood cells by using a panel of prion antibodies", *Transfusion* **42**(5), 517–526.

Beringue, V., Demoy, M., Lasmezas, C.I., Gouritin, B., Weingarten, C., Deslys, J.P., Andreux, J.P., Couvreur, P. and Dormont, D. (2000) "Role of spleen macrophages in the clearance of scrapie agent early in pathogenesis", *J. Pathol.* **190**(4), 495–502.

Borchelt, D.R., Scott, M., Taraboulos, A., Stahl, N. and Prusiner, S.B. (1990) "Scrapie and cellular prion proteins differ in their kinetics of synthesis and topology in cultured cells", *J. Cell Biol.* **110**(3), 743–752.

Borchelt, D.R., Taraboulos, A. and Prusiner, S.B. (1992) "Evidence for synthesis of scrapie prion proteins in the endocytic pathway", *J. Biol. Chem.* **267**(23), 16188–16199.

Bradley, R. (1999) "BSE transmission studies with particular reference to blood", *Dev. Biol. Stand.* **99**, 35–40.

Brandner, S., Klein, M.A. and Aguzzi, A. (1999) "A crucial role for B cells in neuroinvasive scrapie", *Transfus. Clin. Biol.* **6**(1), 17–23.

Brown, K.L., Stewart, K., Ritchie, D.L., *et al.* (1999) "Scrapie replication in lymphoid tissues depends on prion protein-expressing follicular dendritic cells", *Nat. Med.* **5**(11), 1308–1312.

Bueler, H., Aguzzi, A., Sailer, A., *et al.* (1993) "Mice devoid of PrP are resistant to scrapie", *Cell* **73**(7), 1339–1347.

Capellari, S., Zaidi, S.I., Urig, C.B., Perry, G., Smith, M.A. and Petersen, R.B. (1999) "Prion protein glycosylation is sensitive to redox change", *J. Biol. Chem.* **274**(49), 34846–34850.

Cashman, N.R., Loertscher, R., Nalbantoglu, J., *et al.* (1990) "Cellular isoform of the scrapie agent protein participates in lymphocyte activation", *Cell* **61**, 185–192.

Collinge, J. (1997) "Human prion diseases and bovine spongiform encephalopathy (BSE)", *Hum. Mol. Genet.* **6**(10), 1699–1705.

Dodelet, V.C. and Cashman, N.R. (1998) "Prion protein expression in human leukocyte differenciation", *Blood* **91**, 1556–1561.

Dürig, J., Giese, A., Schulz-Schaeffer, W., *et al.* (2000) "Differential constitutive and activation-dependent expresion of prion protein in human peripheral blood leukocytes", *Br. J. Haematol.* **108**, 488–496.

Farquhar, C.F., Dornan, J., Moore, R.C., Somerville, R.A., Tunstall, A.M. and Hope, J. (1996) "Protease-resistant PrP deposition in brain and non-central nervous system tissues of a murine model of bovine spongiform encephalopathy", *J. Gen. Virol.* **775**(Pt8), 1941–1946.

Fournier, J.G. (2001) "Nonneuronal cellular prion protein", *Int. Rev. Cytol.* **208**, 121–160.

Gallo, R.C. and Wong-Staal, F. (1982) "Retroviruses as etiologic agents of some animal and human leukemias and lymphomas and as tools for elucidating the molecular mechanism of leukemogenesis", *Blood* **60**(3), 545–557.

Hill, A.F., Butterworth, R.J., Joiner, S., *et al.* (1999) "Investigation of variant Creutzfeldt-Jakob disease and other human prion diseases with tonsil biopsy samples", *Lancet (N. Am. Ed.)* **353**(9148), 183–189.

Horiuchi, M., Ishiguro, N., Nagasawa, H., Toyoda, Y. and Shinawaga, M. (1997) "Alternative usage of exon 1 of bovine PrP mRNA", *Biochem. Biophys. Res. Commun.* **233**, 650–654.

Huang, F.P., Farquhar, C.F., Mabbott, N.A., Bruce, M.E. and MacPherson, G.G. (2002) "Migrating intestinal dendritic cells transport PrP(Sc) from the gut", *J. Gen. Virol.* **83**(Pt 1), 267–271.

Jimenez-Huete, A., Lievens, P.M., Vidal, R., Piccardo, P., Ghetti, B., Tagliavini, F., Frangione, B. and Prelli, F (1998) "Endogenous proteolytic cleavage of normal and disease-associated isoforms of the human prion protein in neural and non-neural tissues", *Am. J. Pathol.* **153**(5), 1561–1572.

Klein, M.A., Frigg, R., Flechsig, E., *et al.* (1997) "A crucial role for B cells in neuroinvasive scrapie", *Nature* **390**(6661), 687–690.

Klein, M.A., Frigg, R., Raeber, A.J., *et al.* (1998) "PrP expression in B lymphocytes is not required for prion neuroinvasion", *Nat. Med.* **4**(12), 1429–1433.

Krakauer, D.C., Zanotto, P.M. and Pagel, M. (1998) "Prion's progress: patterns and rates of molecular evolution in relation to spongiform disease", *J. Mol. Evol.* **47**, 133–145.

Lascelles, A.K. and McDowell, G.H. (1974) "Localized humoral immunity with particular reference to ruminants", *Transplant Rev.* **19**(0), 170–208.

Lehmann, S. and Harris, D.A. (1997) "Blockade of glycosylation promotes acquisition of scrapie-like properties by the prion protein in cultured cells", *J. Biol. Chem.* **272**(34), 21479–21487.

Li, R., Liu, D., Zanusso, G., *et al.* (2001) "The expression and potential function of cellular prion protein in human lymphocytes", *Cell Immunol.* **207**(1), 49–58.

Liu, T., Li, R., Wong, B.S., *et al.* (2001) "Normal cellular prion protein is preferentially expressed on subpopulations of murine hemopoietic cells", *J. Immunol.* **166**(6), 3733–3742.

Lopez Garcia, F., Zahn, R., Riek, R. and Wuthrich, K. (2000) "NMR structure of the bovine prion protein", *Proc. Natl Acad. Sci. USA* **97**(15), 8334–8339.

Mabbott, N.A., Brown, K.L., Manson, J. and Bruce, M.E. (1997) "T-lymphocyte activation and cellular form of the prion protein", *Immunology* **92**, 161–165.

Mabbott, N.A., Williams, A., Farquhar, C.F., Pasparakis, M., Kollias, G. and Bruce, M.E. (2000) "Tumor necrosis factor alpha-deficient, but not interleukin-6-deficient, mice resist peripheral infection with scrapie", *J. Virol.* **74**(7), 3338–3344.

Mabbott, N.A., McGovern, G., Jeffrey, M. and Bruce, M.E. (2002) "Temporary blockade of the tumor necrosis factor receptor signaling pathway impedes the spread of scrapie to the brain", *J. Virol.* **76**(10), 5131–5139.

MacGregor, I., Drummond, O., Turner, M., Barclay, R. and Prowse, C. (2000) "Distribution of normal prion protein in blood", *Transfus. Sci.* **22**(1–2), 51.

Madec, J.Y., Groschup, M.H., Calavas, D., Junghans, F. and Baron, T. (2000) "Protease-resistant prion protein in brain and lymphoid organs of sheep within a naturally scrapie-infected flock", *Microb. Pathog.* **28**(6), 353–362.

McBride, P.A., Eikelenboom, P., Kraal, G., Fraser, H. and Bruce, M.E. (1992) "PrP protein is associated with follicular dendritic cells of spleens and lymph nodes in uninfected and scrapie-infected mice", *J. Pathol.* **168**(4), 413–418.

Mohri, S., Farquhar, C.F., Somerville, R.A., Jeffrey, M., Foster, J. and Hope, J. (1992) "Immunodetection of a disease specific PrP fraction in scrapie-affected sheep and BSE-affected cattle", *Vet. Rec.* **131**, 537–539.

Montrasio, F., Frigg, R., Glatzel, M., *et al.* (2000) "Impaired prion replication in spleens of mice lacking functional follicular dendritic cells", *Science* **288**(5469), 1257–1259.

Moudjou, M., Frobert, Y., Grassi, J. and la Bonnardiere, C. (2001) "Cellular prion protein status in sheep: tissue-specific biochemical signatures", *J. Gen. Virol.,* **82**(Pt 8), 2017–2024.

Pammer, J., Suchy, A., Rendl, M. and Tschachler, E. (1999) "Cellular prion protein expressed by bovine squamous epithelia of skin and upper gastrointestinal tract", *Lancet (N. Am. Ed.)* **354**(9191), 1702–1703.

Petersen, R.B., Parchi, P., Richardson, S.L., Urig, C.B. and Gambetti, P. (1996) "Effect of the D178N mutation and the codon 129 polymorphism on the metabolism of the prion protein", *J. Biol. Chem.* **271**(21), 12661–12668.

Prinz, M., Montrasio, F., Klein, M.A., Schwarz, P., Priller, J., Odermatt, B., Pfeffer, K. and Aguzzi, A. (2002) "Lymph nodal prion replication and neuroinvasion in mice devoid of follicular dendritic cells", *Proc. Natl Acad. Sci. USA* **99**(2), 919–924.

Prusiner, S.B. (1982) "Novel proteinaceous infectious particles cause scrapie", *Science* **216**, 136–144.

Race, R., Oldstone, M. and Chesebro, B. (2000) "Entry versus blockade of brain infection following oral or intraperitoneal scrapie administration: role of prion protein expression in peripheral nerves and spleen", *J. Virol.* **74**(2), 828–833.

Raeber, A.J., Brandner, S., Klein, M.A., Benninger, Y., Musahl, C., Frigg, R., Roeckl, C., Fisher, M.B., Weissmann, C. and Aguzzi, A. (1998) "Transgenic and knock-out mice in research on prion diseases", *Brain Pathol.* **8**(4), 715–733.

Russelakis-Carneiro, M., Saborio, G.P., Anderes, L. and Soto, C. (2002) "Changes in the glycosylation pattern of prion protein in murine scrapie: implications for the mechanism of neurodegeneration in prion diseases", *J. Biol. Chem.* **277**(39), 36872–36877.

Scott, M.R., Will, R., Ironside, J., *et al.* (1999) "Compelling transgenetic evidence for transmission of bovine spongiform encephalopathy prions to humans", *Proc. Natl Acad. Sci. USA* **96**(26), 15137–15142.

Shyng, S.L., Huber, M.T. and Harris, D.A. (1993) "A prion protein cycles between the cell surface and an endocytic compartment in cultured neuroblastoma cells", *J. Biol. Chem.* **268**(21), 15922–15928.

Somerville, R.A., Birkett, C.R., Farquhar, C.F., *et al.* (1997) "Immunodetection of PrPSc in spleens of some scrapie-infected sheep but not BSE-infected cows", *J. Gen. Virol.* **78**((Pt 9), 2389–2396.

Takeda, K., Tanaka, T., Shi, W., *et al.* (1996) "Essential role of Stat6 in IL-4 signalling", *Nature* **380**(6575), 627–630.

Taraboulos, A., Raeber, A.J., Borchelt, D.R., Serban, D. and Prusiner, S.B. (1992) "Synthesis and trafficking of prion proteins in cultured cells", *Mol. Biol. Cell* **3**(8), 851–863.

Thielen, C., Mélot, F., Jolois, O., *et al.* (2001) "Isolation of bovine follicular dendritic cells allows the demonstration of a particular cellular prion protein", *Cell Tissue Res.* **306**(1), 49–55.

Weissmann, C., Bueler, H., Fischer, M., Sauer, A. and Aguet, M. (1994) "Susceptibility to scrapie in mice is dependent on PrPc", *Philos. Trans. R. Soc. Lond. B. Biol. Sci.* **343**(1306), 431–433.

Wells, G.A., Spencer, Y.I. and Haritani, M. (1994) "Configurations and topographic distribution of PrP in the central nervous system in bovine spongiform encephalopathy: an immunohistochemical study", *Ann. NY Acad. Sci.* **724**, 350–352.

Wells, G.A., Hawkins, S.A., Green, R.B., *et al.* (1998) "Preliminary observations on the pathogenesis of experimental bovine spongiform encephalopathy (BSE): an update", *Vet. Rec.* **142**(5), 103–106.

